# An extension of the RiMAX multipath estimation algorithm for ultra-wideband channel modeling

**DOI:** 10.1186/s13638-018-1177-3

**Published:** 2018-06-27

**Authors:** Brecht Hanssens, Emmeric Tanghe, Davy P. Gaillot, Martine Liénard, Claude Oestges, David Plets, Luc Martens, Wout Joseph

**Affiliations:** 1 INTEC - WAVES, Ghent University - imec, Ghent, Belgium; 2 IEMN/TELICE, University of Lille 1, Lille, France; 3 ICTEAM, Université Catholique de Louvain, Louvain-la-Neuve, Belgium

**Keywords:** Ultra-wideband, Multipath propagation, Multipath estimation, RiMAX, Specular multipath components, Dense multipath components, Channel modeling, Channel sounding, Indoor environment

## Abstract

This work presents an extension of the high-resolution RiMAX multipath estimation algorithm, enabling the analysis of frequency-dependent propagation parameters for ultra-wideband (UWB) channel modeling. Since RiMAX is a narrowband algorithm, it does not account for the frequency-dependency of the radio channel or the environment. As such, the impact of certain materials in which these systems operate can no longer be considered constant with respect to frequency, preventing an accurate estimation of multipath parameters for UWB communication. In order to track both the specular and dense multipath components (SMC and DMC) over frequency, an extension to the RiMAX algorithm was developed that can process UWB measurement data. The advantage of our approach is that geometrical propagation parameters do not appear or disappear from one sub-band onto the next. The UWB-RiMAX algorithm makes it possible to re-evaluate common radio channel parameters for DMC in the wideband scenario, and to extend the well-known deterministic propagation model comprising of SMC alone, towards a more hybrid model containing the stochastic contributions from the DMC’s distributed diffuse scattering as well.

Our algorithm was tested with synthetic radio channel models in an indoor environment, which show that our algorithm can match up to 99% of the SMC parameters according to the multipath component distance (MCD) metric and that the DMC reverberation time known from the theory of room electromagnetics can be estimated on average with an error margin of less than 2 ns throughout the UWB frequency band. We also present some preliminary results in an indoor environment, which indicate a strong presence of DMC and thus diffuse scattering. The DMC power represents up to 50% of the total measured power for the lower UWB frequencies and reduces to around 30% for the higher UWB frequencies.

## Introduction

In the last couple of years, the physical view of how the radio channel is composed has undergone certain changes. The radio channel used to be considered as a collection of specular multipath components (SMC) that have well-defined discrete locations in the different radio channel dimensions (such as the spatial-, frequency-, or time-delay domain). These SMC are the propagation paths which are considered to have a significant influence in the total received power and are comprised of the strongest (specular) reflections. Presently, it is widely accepted that over these dimensions, a part of the radio channel is also continuous, originating mainly from distributed diffuse scattering on electrically small objects [1, 2], which are inherently more present at sub-20 GHz frequencies [3, 4]. These are the so-called dense multipath components (DMC). The ideology behind the DMC is to include all radio channel energy that cannot be associated with the SMC, disregarding whether they originate from reflections, diffractions, etc. This implies that certain (specular) multipath components, from a physical point of view, can be regarded as being part of the DMC process because they cannot be reliably detected. This could be, e.g., due to the limited accuracy of the radio channel model used, a too low signal-to-noise ratio (SNR), or because they lie very close to other multipath components in the time-delay or angular domains [5, 6]. Since the phase of the DMC is inaccessible for estimation, only their average power can be modeled across the aforementioned domains. This opposes the SMC contribution to the channel, where both the power and the phase of each propagation path are accessible.

The objective of this work is to extend the high-resolution DMC-inclusive RiMAX algorithm [7] from its narrowband channel model to an ultra-wideband (UWB) one, facilitating the analysis of frequency-dependent propagation parameters for UWB channel modeling. UWB is currently standardized in wireless personal area network (WPAN) IEEE 802.15.4a, which thanks to its large bandwidth allows for data rates over 2 Gbit/s over a short distance [8]. UWB systems are characterized by their ability to transmit small pulses with a very low power density (limited to – 41.3 dBm/MHz) in a large frequency band (3.1–10.6 GHz) [9]. This enables such systems to harmlessly operate in frequency bands currently occupied by other applications. Combining UWB technology with a MIMO antenna configuration vastly increases the capacity of the UWB system, allowing for extremely high data rates [10], and the accurate localization of target nodes in wireless networks [11–16].

Several ray-tracing studies [17] and measurements [18, 19] have already shown that SMC alone is not sufficient to describe the complicated interactions to which electromagnetic waves are exposed to in a real environment. This is especially true for high-resolution parameter estimation in the field of channel sounding, where the number of resolvable SMC is limited by the overall characteristics of the measurement system, the obtained SNR, and the design of the multipath estimation algorithm [20]. This led to the innovative introduction of DMC in the RiMAX multipath estimation algorithm, to account for parts of the radio channel that cannot be resolved as SMC. However, in contrast to standardized UWB channel models such as [21], the inclusion of the DMC contributions in it are missing, which this work aims to overcome.

Multidimensional channel sounding is a necessary process to describe the geometric structure of the multiple-input multiple-output (MIMO) radio channel in terms of geometrical parameters such as the geometrical parameters such as [22]. These parameters are needed to deduce a geometry-based stochastic channel model (GBSCM) for the evaluation of MIMO transmission systems [23] and to investigate their exact propagation mechanisms. They can also be used to develop localization algorithms for location estimation or tracking. In particular, the double-directional modeling of the radio channel has attracted a lot of interest because it gives a better physical insight into the wave propagation mechanisms in real environments. The effect of DMC has been investigated on the angular properties of the radio channel in [5, 6, 20, 24], the polarization characteristics in [23, 25, 26], the clustering of multipath components in [1, 27], and the channel capacity in [28, 29] for outdoor and industrial scenarios, respectively.

The novelty of our approach is that the newly developed UWB-RiMAX algorithm allows for the global (i.e., frequency-wise) estimation of multipath parameters throughout the UWB frequency band, whilst maintaining the DMC-inclusive behavior of the radio channel. In contrast with executing the RiMAX algorithm in multiple UWB sub-bands, the advantage of our algorithm is that the AoD, AoA, and ToA are kept constant over the entire UWB bandwidth in the initialization procedure, ensuring that geometrical propagation parameters do not appear or disappear from one sub-band onto the next. This makes it possible to estimate the physically most likely radio channel parameters for the SMC and DMC components in the wideband scenario and to extend the well-known deterministic propagation model comprising of SMC alone, towards a hybrid model which also contains the stochastic contributions due to the DMC’s distributed diffuse scattering.

The structure of this paper is as follows; Section 2 describes related work to our approach, whilst Section 3 explains the applied data model. Section 4 then covers the UWB-RiMAX multipath estimation algorithm, and Section 5 describes the applied evaluation metrics. Finally, Sections 6 and 7 highlight the simulation and measurement results of our algorithm, and Section 8 summarizes this paper with a conclusion and some ideas for future work.

## Related work

It was shown previously that the contribution of the DMC to the capacity of the channel in MIMO systems is quite significant, and often larger than that of the SMC. In [29], an experimental analysis of the DMC was conducted in an industrial environment at 3 GHz, where the DMC covariance structure of the RiMAX data model was validated. The authors found that the DMC power accounted for 23 to 70% of the total channel power and found it was more important than in office environments due to its highly cluttered and metallic nature. Similar results are reported in [28] for outdoor environments at 5.2 GHz, which found DMC power contributions ranging from 10% of the total channel power, even up to 90%. Currently, only a few more studies have been conducted regarding the influence of DMC [5, 6, 26, 30, 31]. It was shown in [32, 33] and [34] that the DMC increases the level of the reconstructed eigenvalues, resulting in a better approximation of the measured eigenvalue structure of the MIMO channel. This indicates that an accurate modeling of the DMC parameters is necessary to prevent the underestimation of the MIMO transmission performance [28].

Naturally, the introduction of DMC in the physical model of the radio channel means that common radio channel parameters have to be re-evaluated for diffuse scattering as well [26]. This includes parameters such as mean delay, delay spread, Ricean K-factor, shadowing, fading, cross polarization and ratios. Recent studies such as [26] found that the SMC and DMC power show, on average, a strong correlation of about 0.90 and 0.95 for line-of-sight (LoS) and obstructed-line-of- sight (OLoS) scenarios, respectively. This implies that the DMC can alternatively be interpreted as the non-coherent superposition of paths with weaker SNR, which still follow the specular power decay as a function of distance [5, 6]. The same study found that for OLoS scenarios, the cross-polar normalized DMC power even exceeds 60% on average, indicating that these channels could be modeled relatively accurately by only considering their DMC characteristics (as it is done in the original room electromagnetics model [35]). Hence, simple DMC models can be used to design more advanced channel models, as e.g. proposed in [36], where a distance-dependent model for the power delay profile (PDP) of in-room radio channels was developed. The PDP in this model assumes an early primary component and a DMC reverberant component responsible for the shaping of the tail in the PDP.

The physical reality of DMC raises the question of how well estimation algorithms which historically did not include DMC in their signal model, such as estimation of signal parameters via rotational invariance techniques (ESPRIT) [37] or space-alternating generalized expectation-maximization (SAGE) [38], can estimate the SMC part of the channel. This was investigated in [39, 40], in which the authors compared both ESPRIT and SAGE to the performance of the DMC-inclusive RiMAX algorithm. The results of this study demonstrated that SMC estimation in the presence of DMC is prone to large errors if the signal model is not accordingly modified to cope with DMC contributions, as is the case with ESPRIT or SAGE. This was also shown theoretically in [41]. Therefore, determining the DMC by simply subtracting the specular part (estimated by ESPRIT or SAGE) from the total channel response is flawed and must be avoided. For a reliable estimation of the SMC and/or DMC parameters, the use of DMC-inclusive algorithms such as RiMAX is highly recommended. Next to that, since the resolution and accuracy of classical signal processing algorithms is limited by the available measurement aperture in the space-frequency-time domain, parametric super-resolution algorithms such as ESPRIT, SAGE, and RiMAX are more suitable to enhance the time-delay resolution. This is done by fitting an appropriate data model to the measured data, allowing the algorithm to overcome the Fourier limitation of the delay resolution.

In [42, 43], the UWB-SAGE algorithm was proposed, which is an extension to the SAGE channel estimation algorithm for UWB channel modeling. The UWB-SAGE algorithm estimates a certain number of individual propagation paths from the measured data and estimates the AoD, AoA, ToA, and the variation of the amplitude and phase for each path. UWB-SAGE is based on the assumption that the UWB channel can be expressed as the superposition of a certain number of sub-bands, in which the scattering loss and the antenna directivity is sufficiently constant. The log-likelihood of the whole UWB bandwidth is then defined as the sum of the log-likelihoods of its sub-bands. This process reduces the distortion effect of amplitude and phase caused by antennas when the parameters of the incident waves are estimated. However, at the same time, the resolution of time-delay decreases due to the sub-band processing, making it necessary to appropriately choose the bandwidth of the sub-bands in which the total UWB bandwidth is divided. However, this algorithm disregards the influence of DMC on the channel transfer function, and is thus not an appropriate algorithm for channel parameter estimation or channel modeling. In our work, the ideology of the UWB-SAGE algorithm will be incorporated in the DMC-inclusive RiMAX algorithm.

## Channel model

### Specular- and dense multipath components

To describe the geometric properties of the electromagnetic waves of the MIMO propagation channel in terms of AoD, AoA, and ToA, multidimensional frequency domain channel sounding must be performed. This can be done with (virtual) MIMO array systems, consisting of *M*_*T*_ and *M*_*R*_ antennas at transmitter (Tx) and receiver (Rx), sampled at *M*_*f*_ frequency points. As such, the total amount of samples can be defined by *M* as follows:

1 $$ M = M_{T} M_{R} M_{f}.  $$

An observation of the frequency response of a MIMO radio channel ***h*** can be modeled as the superposition of a deterministic part ***x***(***θ***_smc_) (determined by the SMC parameter set ***θ***_smc_) and a stochastic part ***d***(***θ***_dan_) (diffuse scattering and noise; determined by the DMC and noise (DAN) parameter set ***θ***_dan_). Both parameter sets will be described later in this section.

2 $$ \begin{aligned} \boldsymbol{h} &=\boldsymbol{x}(\boldsymbol{\theta}_{\text{smc}}) + \boldsymbol{d}(\boldsymbol{\theta}_{\text{dan}})\\ \boldsymbol{h} & \in \mathbb{C}^{M \times 1}. \end{aligned}  $$

The deterministic part ***x***(***θ***_smc_) of the data model acts as the first order statistics of the radio channel, so that it can be interpreted as the mean of ***h***, whilst the stochastic part ***d***(***θ***_dan_) describes the second-order statistics by means of its covariance matrix $\boldsymbol {R}(\boldsymbol {\theta }_{\text {dan}}) \in \mathbb {C}^{M \times M}$, which will be discussed in Section 5.1.2. A realization of the radio channel ***h*** can be considered as a random variable distributed according to a complex multivariate Gaussian distribution $\boldsymbol {h} \sim \mathcal {N}_{c} (\boldsymbol {x}(\boldsymbol {\theta }_{\text {smc}}), \boldsymbol {R}(\boldsymbol {\theta }_{\text {dan}}))$ [44] as follows:

3 $$ p(\boldsymbol{h}) = \frac{1}{\pi^{M} \det(\boldsymbol{R})} e^{-(\boldsymbol{h}-\boldsymbol{x})^{H} \boldsymbol{R}^{\text{-1}} (\boldsymbol{h}-\boldsymbol{x})}.  $$

An estimate of the most likely SMC and DMC and noise parameters can be found by maximizing the likelihood function of Eq. (3). Since this is not a trivial task, estimation frameworks such as the RiMAX algorithm will estimate $\hat {\boldsymbol {\theta }}_{\text {smc}}$ and $\hat {\boldsymbol {\theta }}_{\text {dan}}$ of the deterministic and stochastic arrays, such that they maximize the likelihood of observing the measured frequency response ***h*** of the radio channel. The objective is thus to find the parameters $\hat {\boldsymbol {\theta }}_{\text {smc}}$ and $\hat {\boldsymbol {\theta }}_{\text {dan}}$ that maximize the correlation with the measurement data. A maximum likelihood (ML) estimator for the parameters ***θ***_smc_ and ***θ***_dan_ has been proposed in [7, 22], exploiting the fact that the parameters of the two components of the channel model are asymptotically independent. Therefore, one can decouple the estimation problem into two separate estimation problems. The resulting RiMAX algorithm is iterative and alternates between the maximization of the likelihood function with respect to the parameters ***θ***_smc_ and ***θ***_dan_. It has an approximately linear computational complexity in the number of propagation paths *P* and in the number of data samples *M* [7].

Based on the capability of the RiMAX algorithm to extract both parameter sets from the (virtual) array measurement data, the following structures for the deterministic and stochastic parameter sets ($\boldsymbol {\theta }_{\text {smc}} \in \mathbb {C}^{P \times 4S}$ and $\boldsymbol {\theta }_{\text {dan}} \in \mathbb {R}^{4 \times S}$, respectively) can be adopted:

4 $$\begin{array}{*{20}l} \boldsymbol{\theta}_{\text{smc}} & = \left[ \begin{array}{c} \boldsymbol{\varphi_{D}}^{T} \\ \boldsymbol{\varphi_{A}}^{T} \\ \boldsymbol{\tau_{A}}^{T} \\ \boldsymbol{\gamma}^{T} \end{array}\right]^{T} \enskip \begin{array}{l} \leftarrow \text{SMC angle of departure [rad]} \\ \leftarrow \text{SMC angle of arrival [rad]} \\ \leftarrow \text{SMC time-delay of arrival [s]} \\ \leftarrow \text{SMC complex amplitude [/].} \end{array} \end{array} $$

In (4), ***φ***_***D***_, ***φ***_***A***_, ***τ***_***A***_, and ***γ*** are *P*×*S* matrices, where *P* is the number of SMCs extracted from the measurement data, and *S* is the number of sub-bands in which the total UWB bandwidth was partitioned (in analogy with the UWB-SAGE algorithm). As such, ***θ***_smc_ is of size 4*S*×*P*. We will discuss the frequency-dependency of the geometrical parameters of the propagation paths in Section 4.2. Each row in the aforementioned matrices ***φ***_***D***_, ***φ***_***A***_, and ***τ***_***A***_ contains the corresponding specular parameter for each of the $p \in \mathcal {P}$ propagation paths ($P = |\mathcal {P}|$ in total) and describes its frequency-dependency in each of the $s \in \mathcal {S}$ sub-bands ($S = |\mathcal {S}|$ in total). We note that the angular modeling was limited to that of the azimuthal plane, which is acceptable since most measurement campaigns are only performed with a planar array, with which the estimation of elevation parameters is not possible. The extension of the data model to the elevation domain is straightforward, where the AoD and AoA of the SMC will now have an extra elevation component together with an azimuthal component. We also consider only a single snapshot of the channel, such that the covariance matrix ***R***(***θ***_dan_) is only averaged over one observation of the channel. Whilst it would be more reliable to use several snapshots of the channel in a real measurement environment, we would then have to impose a parametric model to handle the time dependence of the SMC, or assume them to be independent and independent and identically distributed (i.i.d.) across snapshots in time. Since this is out of the scope for the purpose of this paper, we will leave this up for future work.

5 $$\begin{array}{*{20}l} \boldsymbol{\theta}_{\text{dan}} & = \left[ \begin{array}{c} \boldsymbol{\alpha_{1}} \\ \boldsymbol{\tau_{d}} \\ \boldsymbol{\tau_{r}} \\ \boldsymbol{\alpha_{0}} \end{array}\right] \enskip \begin{array}{l} \leftarrow \text{DMC peak power [W]} \\ \leftarrow \text{DMC onset time [s]} \\ \leftarrow \text{DMC reverberation time [s]} \\ \leftarrow \text{Noise power [W].} \\ \end{array} \end{array} $$

In (5), ***θ***_dan_ contains the DMC parameters $\left (\left [\boldsymbol {\alpha _{1}} \in \mathbb {R}^{1 \times S}, \boldsymbol {\tau _{d}} \in \mathbb {R}^{1 \times S}, \boldsymbol {\tau _{r}} \in \mathbb {R}^{1 \times S}\right ]\right)$ and the noise parameters ($\boldsymbol {\alpha _{0}} \in \mathbb {R}^{1 \times S}$) for each sub-band $s \in \mathcal {S}$. A discussion of the model for the DMC can be found in [7]. The model is based on the observation that the PDP *ψ*(*τ*) [W] of the DMC and noise, which describes how the power of a signal is distributed over the time-delay domain, has a base time-delay *τ*_*d*_ related to the distance between the transmitter and receiver, together with an exponential decay over time-delay (see Eq. (6)), corrupted by complex additive white Gaussian noise with power *α*_0_:

6 $$  \psi (\tau) = \left\{ \begin{array}{ll} \alpha_{1} \hspace{1mm} e^{\left(- \frac{\tau-\tau_{d}}{\tau_{r}} \right)}+\alpha_{0}, & \text{if } \tau > \tau_{d}\\ \alpha_{0}, & \text{otherwise}. \end{array}\right.  $$

In Eq. (6), *α*_1_, *τ*_*d*_, *τ*_*r*_, and *α*_0_ are the four parameters which fully describe the DMC and noise characteristics of each sub-band and are gathered in the DMC and noise parameter vector ***θ***_dan_. In this work, we will assume that the DMC is spatially white at the transmit and receive side of the measurement system, meaning that they have constant angular power densities. It should be noted that recent works will assume the DMC to be spatially correlated with the SMC. For example, [25, 45] report a correlation between the location of SMC and DMC in the angular domain. In [27], the DMC is modeled as local clusters around the SMC. In [20, 46], it is proposed that the Power Angular Profile (PAP) is to be modeled by a uni-modal Von-Mises distribution.

The covariance matrix ***R***(***θ***_dan_) can be constructed by converting the model in Eq. (6) from the time-delay domain to the frequency domain. To do so, $\boldsymbol {\kappa }(\boldsymbol {\theta }_{\text {dan}}) \in \mathbb {C}^{N_{F} \times 1}$ [W] is first introduced, which denotes a band-limited sampled version of the Fourier transform of Eq. (6), and can be defined for a bandwidth *B* and *M*_*f*_ frequency samples as follows:

7 $$ {\begin{aligned} {}\boldsymbol{\kappa}(\boldsymbol{\theta}_{\text{dan}}) =& \alpha_{0}~\boldsymbol{e_{0}} + \frac{\alpha_{1}}{M_{f}} \left[\frac{1}{\beta_{d}}, \frac{e^{-j2\pi \tau_{d}}}{\beta_{d} + j2\pi\frac{1}{M_{f}}} \cdots \right.\\& \qquad \qquad\qquad \qquad \qquad \quad \left. \frac{e^{-j2\pi \tau_{d} \left(M_{f}-1\right)}}{\beta_{d} + j2\pi\frac{\left(M_{f}-1\right)}{M_{f}}}\right], \end{aligned}}  $$

in which $\boldsymbol {e_{0}} \in \mathbb {N}^{M_{f} \times 1}$ is a unit vector defined as follows:

8 $$ \boldsymbol{e_{0}} = \left[1,0, \cdots, 0\right].  $$

Furthermore, in Eq. (7), *β*_*d*_ is the normalized coherence bandwidth (dimensionless), calculated as follows:

9 $$ \beta_{d} = \frac{B_{d}}{B} = \frac{1}{B \cdot \tau_{r}},  $$

in which *B*_*d*_ is the coherence bandwidth (Hz), normalized by the bandwidth per sub-band *B* (Hz). Furthermore, the frequency sampling interval *Δ*_*f*_ (Hz) can be written as a function of *B* and *M*_*f*_ as follows:

10 $$ \Delta_{f} = \frac{B}{M_{f}-1}.  $$

After calculating ***κ***(***θ***_dan_), the covariance matrix ***R***(***θ***_dan_) of the DMC and noise can be calculated by applying the Toeplitz-operator [7] as follows:

11 $$ \boldsymbol{R}(\boldsymbol{\theta}_{\text{dan}}) = \text{toep} \left(\boldsymbol{\kappa}(\boldsymbol{\theta}_{\text{dan}}), \boldsymbol{\kappa}(\boldsymbol{\theta}_{\text{dan}})^{H}\right).  $$

The original data model of the RiMAX estimation algorithm follows the narrowband assumption, hence stating that the SMC and DMC are Kronecker-separable in the spatial and frequency domains in order to keep the algorithm computationally viable [7]. In our measurements section, prior to the processing of the measurement data, we will check for the uncorrelated scattering assumption, which needs to hold in order to apply the Kronecker model. For a large MIMO configuration in a given communication system, the dimensions of the covariance matrix ***R***(***θ***_dan_) becomes too large to allow for a reliable estimation (remember that it has a size of [*M*×*M*]). This becomes a processing burden to accurately model the interaction between transmitter and receiver, so that both ends of a communication system need to be decoupled by applying the well-known Kronecker model. We refer to [47] for a discussion of its applicability and limitations.

In the Kronecker model, the covariance matrices at transmitter and receiver are assumed independent and separable, allowing the full covariance matrix of the channel to be expressed as the Kronecker-product of several smaller matrices. Thus, the covariance matrix ***R*** is assumed to have the following structure [7]:

12 $$ \boldsymbol{R} = \boldsymbol{R}_{T} \otimes \boldsymbol{R}_{R} \otimes \boldsymbol{R}_{f} + \alpha_{0} \boldsymbol{I}_{M},  $$

in which $\boldsymbol {R}_{T} \in \mathbb {C}^{M_{T} \times M_{T}}$ and $\boldsymbol {R}_{R} \in \mathbb {C}^{M_{R} \times M_{R}}$ are the covariance matrices at the transmitter and receiver, respectively, describing the spatial distribution of the DMC at both ends, whereas $\boldsymbol {R}_{f} \in \mathbb {C}^{M_{f} \times M_{f}}$ is the covariance matrix in the frequency domain. The term *α*_0_***I*** denotes the amount of complex additive white Gaussian measurement noise, which contributes to the stochastic part of the data model. The matrix ***I***_*M*_ is an identity matrix of size [*M*×*M*].

### Modeling propagation paths

In this extension of the RiMAX algorithm, we will assume that the transmitter and receiver are equipped with a uniform circular array (UCA), given that its angular resolution capability is uniform since the effective aperture does not change with azimuth angle. The extension to other array configurations is straightforward since only the steering matrices in the following subsection have to be adjusted. It should be noted that other configurations for the antenna array can be used in this algorithm as well, without much modification to the hereinafter explained matrices. The broadband MIMO radio channel model can be expressed in matrix notation to map the time-delay and angles of a propagation path to its complex notation in the frequency domain. We will therefore define the matrix $\boldsymbol {B}_{f}(\boldsymbol {\tau }_{A}) \in \mathbb {C}^{M_{f} \times P}$ (dimensionless), which maps the time-delays ***τ***_*A*_ of each propagation path $p \in \mathcal {P}$ to its related complex exponential $\phantom {\dot {i}\!}e^{-j2\pi \cdot m \Delta _{f} \cdot \tau _{A,p}}$ notation as follows:

13 $$ {} \boldsymbol{B}_{f}(\boldsymbol{\tau}_{A})\! =\! \left[ \begin{array}{ccc} e^{-j2\pi\Delta_{f} \left(-\frac{M_{f}-1}{2}\right)\tau_{A,1}} & \cdots & e^{-j2\pi\Delta_{f}\left(-\frac{M_{f}-1}{2}\right)\tau_{A,P}} \\ \vdots & \ddots & \vdots \\ e^{-j2\pi\Delta_{f} \left(+\frac{M_{f}-1}{2}\right)\tau_{A,1}} & \cdots & e^{-j2\pi\Delta_{f}\left(+\frac{M_{f}-1}{2}\right)\tau_{A,P}} \end{array}\right],  $$

Similarly, we can define the mapping of both the departing and the arriving angles ***φ***_*D*_ and ***φ***_*A*_ to the transmitting and receiving array responses $\boldsymbol {B}^{s}_{T}(\boldsymbol {\varphi }_{D}) \in \mathbb {C}^{M_{T} \times P}$ (dimensionless) and $\boldsymbol {B}^{s}_{R}(\boldsymbol {\varphi }_{A}) \in \mathbb {C}^{M_{R} \times P}$ (dimensionless) in each sub-band $s \in \mathcal {S}$ as follows:

14 $$ {} \boldsymbol{B}^{s}_{T}(\boldsymbol{\varphi}_{D})\! =\! \left[\! \begin{array}{ccc} e^{-j\frac{2\pi}{\lambda_{s}}r\cos(\varphi_{D,1}-\rho_{1})} & \cdots \!& \! e^{-j\frac{2\pi}{\lambda_{s}}r\cos(\varphi_{D,P}-\rho_{1})} \\ \vdots & \ddots & \vdots \\ e^{-j\frac{2\pi}{\lambda_{s}}r\cos\left(\varphi_{D,1}-\rho_{M_{T}}\right)} & \cdots & e^{-j\frac{2\pi}{\lambda_{s}}r\cos\left(\varphi_{D,P}-\rho_{M_{T}}\right)} \end{array}\right],  $$

and

15 $$ {{} \begin{aligned} \boldsymbol B^{s}_{R}(\boldsymbol{\varphi}_{A}) \,=\, \left[\! \begin{array}{ccc} e^{-j\frac{2\pi}{\lambda_{s}}r\cos(\varphi_{A,1}-\rho_{1})} & \cdots \!& e^{-j\frac{2\pi}{\lambda_{s}}r\cos(\varphi_{A,P}-\rho_{1})} \\ \vdots & \ddots & \vdots \\ e^{-j\frac{2\pi}{\lambda_{s}}r\cos\left(\varphi_{A,1}-\rho_{M_{R}}\right)} & \cdots & e^{-j\frac{2\pi}{\lambda_{s}}r\cos\left(\varphi_{A,P}-\rho_{M_{R}}\right)} \end{array}\!\right], \end{aligned}}  $$

with *r* being the radius of the UCA, *λ*_*s*_ (m) the wavelength in each sub-band $s \in \mathcal {S}$, and the vector function $\boldsymbol {\rho }(M_{T/R}) \in \mathbb {R}^{M_{T/R} \times 1}$ (rad) mapping the angle between each antenna in the UCA and a chosen reference axis, defined as follows:

16 $$ \boldsymbol{\rho}(M_{T/R}) = (0:1:M_{T/R}-1)\frac{2\pi}{M_{T/R}}.  $$

Both matrices ***B***_*T*_(***φ***_*D*_) and ***B***_*R*_(***φ***_*A*_) describe the complex frequency-dependent far-field beam pattern at each antenna array port at the transmitting and receiving array, respectively.

Now let us consider a measurement snapshot of the multidimensional UWB-MIMO channel ***h***. This snapshot can be defined as the instantaneous frequency domain transfer function of the channel between each MIMO antenna pair and is modeled as a superposition of *P* discrete paths ***x*** plus a contribution of DMC and noise, written as follows:

17 $$ \boldsymbol{h} = \sum\limits_{p=1}^{P} \boldsymbol{x}_{p}\left(\boldsymbol{\theta}_{\text{smc},p}\right) + \boldsymbol{d}(\boldsymbol{\theta}_{\text{dan}}).  $$

The model for a single propagation path *p* in sub-band *s* is given by:

18 $$ \boldsymbol{x}^{s}_{p}\left(\boldsymbol{\theta}^{s}_{\text{smc},p}\right) = \texttt{vec} \left(\boldsymbol{B}_{T}^{s}(\varphi_{D,p}) \Diamond \boldsymbol{B}_{R}^{s}(\varphi_{A,p}) \Diamond \boldsymbol{B}_{f}(\tau_{A,p})\right) \gamma^{s}_{p},  $$

where the operator ♢ denotes Khatri-Rao (column-wise Kronecker) product and the variable $\gamma ^{s}_{p}$ denotes the complex amplitude of path *p* in sub-band *s*. The superposition of *P* paths in sub-band *s* can then be written as follows:

19 $$ \begin{aligned} \boldsymbol{x}^{s}\left(\boldsymbol{\theta}^{s}_{\text{smc}}\right) &= \sum\limits_{p=1}^{P} \boldsymbol{x}^{s}_{p}\left(\boldsymbol{\theta}^{s}_{\text{smc},p}\right) \\ &= \left(\boldsymbol{B}^{s}_{T}(\boldsymbol{\varphi}_{D}) \Diamond \boldsymbol{B}^{s}_{R}(\boldsymbol{\varphi}_{A}) \Diamond \boldsymbol{B}_{f}(\boldsymbol{\tau}_{A})\right) \boldsymbol{\gamma}^{s} \\ &= \boldsymbol{B}^{s}\left(\boldsymbol{\varphi}_{D},\boldsymbol{\varphi}_{A},\boldsymbol{\tau}_{A}\right) \boldsymbol{\gamma}^{s} \in \mathbb{C}^{M \times 1}, \end{aligned}  $$

in which ***B***^*s*^(***φ***_*D*_,***φ***_*A*_,***τ***_*A*_) represents the steering vector in space and frequency for the entire array, and for all propagation paths, in sub-band *s*.

## Extension to UWB-RiMAX algorithm

### Global overview of the algorithm

In a wideband scenario, the reflection coefficients of certain materials in the environment (e.g., furniture, cabinets) can no longer be considered constant with respect to frequency. In addition, the antenna array responses of transmitter and receiver will no longer be frequency-independent and can vary significantly over the total UWB bandwidth. To overcome this issue, the total UWB band was split up into *S* sub-bands to assure frequency stationarity in each sub-band. This is in agreement with the multiband radio UWB principle [48], where the total UWB band is split into multiple sub-bands that are separately processed by the receiver, to avoid problems with the restrictions on the analog RF circuit designs. Although the geometrical propagation characteristics (AoD, AoA, and ToA) are frequency-independent, the complex amplitude of each path will vary throughout the UWB frequency band. Hence, we can adopt the narrowband assumption in each sub-band, making it sufficient in terms of measurement accuracy to describe the directional characteristics of the antenna arrays at the center frequency of each sub-band.

**Fig. 1:**
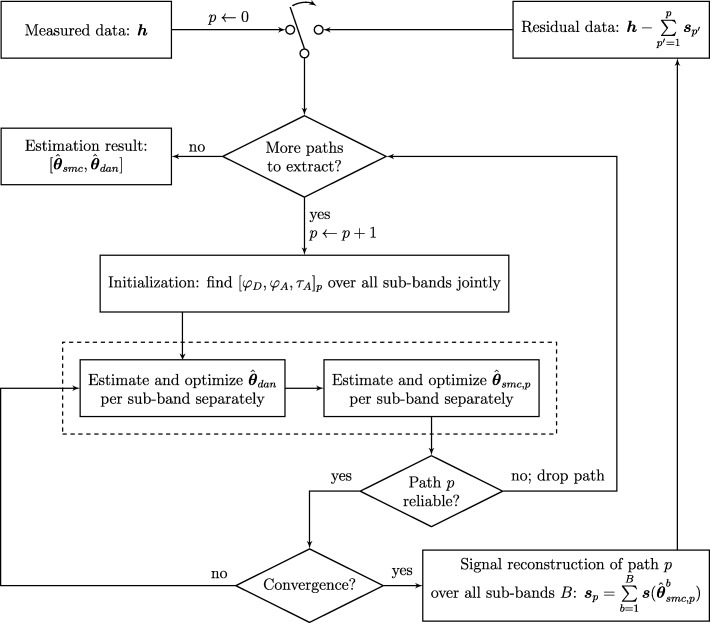
Flowchart of the UWB-RiMAX multipath estimation algorithm. In the first iteration, the input data will be the measured channel ***h***. The one-time switch at the top will ensure that subsequent iterations of the algorithm will make use of the residual channel as input data

In order to properly estimate propagation parameters over the UWB frequency band, an extension to the RiMAX algorithm was developed that can process UWB measurement data and will be referred to as UWB-RiMAX from now on. The global outline for this algorithm is broadly described in Fig. 1. In the next following subsections, we will go deeper into certain aspects of the algorithm.

### Initialization (Fig. 1): modeling frequency-dependency

In this subsection, we will discuss how the frequency-dependency of the SMC was modeled in the initialization procedure. As outlined in Fig. 1, the geometrical parameters ***φ***_***D***_, ***φ***_***A***_, and ***τ***_***A***_ are kept constant over frequency, ensuring that these could not appear or disappear from one sub-band onto the next. In a latter part of the algorithm (estimation and optimization of the SMC (see Fig. 1), these geometrical parameters will be optimized in each sub-band separately, in order not to over-constrain the optimization procedure of the complex amplitudes ***γ***. This also facilitates the optimization, as there is no strong dependency to be modeled between the sub-bands. Doing so, the geometrical parameters can thus vary slightly over frequency after the optimization procedure is carried out, but jointly estimating them in the initialization procedure ensures that they start their optimization from the same set of values.

#### Maximum likelihood expression

The parameter estimation for propagation paths is initialized by a successive interference cancelation type of grid search, where the detection of paths is based on a single path ML criterion [42]. Taking the logarithm of Eq. (3) and maximizing it with respect to the deterministic and stochastic part gives the following ML criterion:

20 $$ \begin{aligned} \left[\begin{array}{l} \hat{\boldsymbol{\theta}}_{\text{smc}} \\ \hat{\boldsymbol{\theta}}_{\text{dan}} \end{array}\right] = & {\underset{\boldsymbol{\theta}_{\text{smc}},\boldsymbol{\theta}_{\text{dan}}}{\arg\max}} \left(-\ln(\det(\boldsymbol{R}(\boldsymbol{\theta}_{\text{dan}}))) \right.\\ &\left. -\left(\boldsymbol{h}-\boldsymbol{x}(\boldsymbol{\theta}_{\text{smc}})\right)^{H} {\boldsymbol{R}}(\boldsymbol{\theta}_{\text{dan}}) (\boldsymbol{h}-\boldsymbol{x}(\boldsymbol{\theta}_{\text{smc}}))\right)\!. \end{aligned}  $$

The parameter estimates $\hat {\boldsymbol {\theta }}_{\text {smc}}$ and $\hat {\boldsymbol {\theta }}_{\text {dan}}$ of the deterministic and stochastic arrays are defined as the maximizing arguments of the nonlinear objective function in Eq. (20). The estimation algorithm determines these parameters such that they maximize the likelihood of observing the measured frequency response ***h*** of the radio channel. Since the number of parameters that are nonlinear in this equation is quite large, the solution to this equation is far from easy since it is not practical to perform a multidimensional search for the joint maximization of this log-likelihood function. By splitting the problem into several smaller sub-problems, and choosing several parameter subsets, it becomes practically feasible to solve the joint maximization problem. We then have to maximize the objective function by alternating between the optimization procedures with respect to these subsets. It is evident that we choose ***θ***_smc_ and ***θ***_dan_ for both these parameter subsets. This approach exploits the fact that the parameters of the two components of the channel model are asymptotically independent, so that one can decouple the full estimation problem into two separate estimation problems. If we assume the covariance matrix ***R***(***θ***_dan_) of the stochastic process is known (thus the parameters ***θ***_dan_ are known), the maximization problem of Eq. (20) reduces to:

21 $$ {} \hat{\boldsymbol{\theta}}_{\text{smc}} = {\underset{\boldsymbol{\theta}_{\text{smc}}}{\arg\min}} \left((\boldsymbol{h}-\boldsymbol{x}(\boldsymbol{\theta}_{\text{dan}}))^{H} {\boldsymbol{R}}(\boldsymbol{\theta}_{\text{dan}}) (\boldsymbol{h}-\boldsymbol{x}(\boldsymbol{\theta}_{\text{smc}}))\right).  $$

The term (***h***−***x***)^*H*^***R***(***h***−***x***) in this equation is the so-called Mahalanobis norm [49]. The ML function in Eq. (21) can be regarded as a nonlinear weighted least squares problem since it is nonlinear in the structural parameters ***θ***_smc_. More specifically, it is an optimally weighted least squares problem since there is no arbitrary weighting matrix used, but instead, we apply the inverse noise covariance matrix ***R***(***θ***_dan_) as weighting matrix. Since the Mahalanobis norm is a non-convex function of the structural parameters ***x***, multiple solutions to this problem exist, and no closed-form solution is available. Therefore, an iterative procedure has to be followed in order to find the ML parameter sets $\hat {\boldsymbol {\theta }}_{\text {smc}}$ and $\hat {\boldsymbol {\theta }}_{\text {dan}}$ (denoted with a hat-operator) of the “true” values of ***θ***_smc_ and ***θ***_dan_, such that they maximize the likelihood of observing the measured frequency response ***h*** of the radio channel given these parameters, as will be discussed in Section 4.3.2.

If the stochastic part of the MIMO channel observation is a zero-mean circular Gaussian i.i.d. process with a covariance matrix equal to *σ*^2^***I***, then the minimization problem is reduced to a classical nonlinear least squares problem [50]. It then becomes a matter of searching for the value $\hat {\boldsymbol {\theta }}_{\text {smc}}$ that minimizes the error ***h***−***x***(***θ***_smc_). This can also be seen as minimizing the Euclidean norm (the so-called Frobenius norm):

22 $$ \hat{\boldsymbol{\theta}}_{\text{smc}} = {\underset{\boldsymbol{\theta}_{\text{smc}}}{\arg\min}} \lVert \boldsymbol{h}-\boldsymbol{x}(\boldsymbol{\theta}_{\text{smc}}) \rVert^{2}_{_{F}}.  $$

#### Global estimation of SMC parameters

Using the general structure of the data model as given by Eq. (18), which describes the contribution of the specular propagation paths to the channel in a certain sub-band $s \in \mathcal {S}$, we can substitute this term in Eq. (19) as follows:

23 $$\begin{array}{*{20}l} {}\left[\begin{array}{l} \hat{\boldsymbol{\theta}}^{s}_{\text{smc}} \\ \hat{\boldsymbol{\theta}}^{s}_{\text{dan}} \end{array}\right] = \underset{\boldsymbol{\theta}^{s}_{\text{smc}},\boldsymbol{\theta}^{s}_{\text{dan}}}{\arg\min} &\left(\left(\boldsymbol{h}^{s}-\boldsymbol{B}^{s}\left(\boldsymbol{\varphi}_{D},\boldsymbol{\varphi}_{A},\boldsymbol{\tau}_{A}\right) \boldsymbol{\gamma}^{s}\right)^{H} \right.\\ &\left. {\boldsymbol{R}}\left(\boldsymbol{\theta}^{s}_{\text{dan}}\right) \left(\boldsymbol{h}^{s}\,-\,\boldsymbol{B}^{s}\left(\boldsymbol{\varphi}_{D},\boldsymbol{\varphi}_{A},\boldsymbol{\tau}_{A}\right) \boldsymbol{\gamma}^{s}\right){\vphantom{\left(\left(\boldsymbol{h}^{s}-\boldsymbol{B}^{s}(\boldsymbol{\varphi}_{D},\boldsymbol{\varphi}_{A},\boldsymbol{\tau}_{A}) \boldsymbol{\gamma}^{s}\right)^{H} \right.}}\right),  \end{array} $$

in which ***h***^*s*^ denotes the frequency response of the MIMO radio channel ***h*** in sub-band *s*, which corresponds to the following range of frequency samples in ***h***: $\left [(s-1)~\frac {M_{f}-1}{S}+1:s~\frac {M_{f}-1}{S}+1\right ]$. Since the complex amplitudes ***γ***^*s*^ are linear in Eq. (23), this minimization problem can be solved directly for $\hat {\boldsymbol {\gamma }}^{s}$ given a parameter set $\hat {\boldsymbol {B}}^{s} = \boldsymbol {B}^{s}\left (\boldsymbol {\varphi }_{D},\boldsymbol {\varphi }_{A},\boldsymbol {\tau }_{A}\right)$. For any $\hat {\boldsymbol {B}}^{s}$, and $\boldsymbol {R}^{s} = \boldsymbol {R}\left (\boldsymbol {\theta }^{s}_{\text {dan}}\right)$, the best linear unbiased estimate (BLUE) is given as follows:

24 $$ \hat{\boldsymbol{\gamma}}^{s} = {\left(\left(\boldsymbol{B}^{s}\right)^{H} {\left(\boldsymbol{R}^{s}\right)} \boldsymbol{B}^{s}\right)} \left(\boldsymbol{B}^{s}\right)^{H} {\left(\boldsymbol{R}^{s}\right)} \boldsymbol{h}.  $$

Inserting Eq. (24) in Eq. (23) yields the following expression for the ML criterion in sub-band *s* [7]:

25 $$ C\left(\boldsymbol{\varphi}_{D},\boldsymbol{\varphi}_{A},\boldsymbol{\tau}_{A},\boldsymbol{h}^{s}\right) = \left| \left(\boldsymbol{B}^{s}_{p}\right)^{H} \boldsymbol{h}^{s} \right|^{2},  $$

in which $\boldsymbol {B}^{s}_{p}$ can be written as follows:

26 $$ \boldsymbol{B}^{s}_{p} = \texttt{vec} \left(\boldsymbol{B}_{T}^{s}(\varphi_{D,p}) \Diamond \boldsymbol{B}_{R}^{s}(\varphi_{A,p}) \Diamond \boldsymbol{B}_{f}(\tau_{A,p})\right).  $$

For the UWB-RiMAX approach, this extends to:

27 $$\begin{array}{*{20}l} C\left(\boldsymbol{\varphi}_{D},\boldsymbol{\varphi}_{A},\boldsymbol{\tau}_{A},\left\{\boldsymbol{h}^{s}\right\}^{S}_{s=1}\right) &= \sum\limits_{s = 1}^{S} C\left(\boldsymbol{\varphi}_{D},\boldsymbol{\varphi}_{A},\boldsymbol{\tau}_{A},\boldsymbol{h}^{s}\right) \end{array} $$

28 $$\begin{array}{*{20}l} &= \sum\limits_{s = 1}^{S} \left| \left(\boldsymbol{B}^{s}_{p}\right)^{H} \boldsymbol{h}^{s} \right|^{2}. \end{array} $$

We can see that this ML criterion estimates the parameters of the electromagnetic waves which extract the largest power from the measured channel ***h***. As such, this approach can allow for a path occurring at a low frequency, but not surviving at a high frequency. In our results, this gives rise to that path having a significant power at the low frequency, but a negligible power at a high frequency. Thus, we can rewrite this minimization procedure in the following three-dimensional simultaneous search:

29 $$ \left(\hat{\boldsymbol{\varphi}_{D}},\hat{\boldsymbol{\varphi}_{A}},\hat{\boldsymbol{\tau}_{A}}\right) = {\underset{\left[\boldsymbol{\varphi}_{D},\boldsymbol{\varphi}_{A},\boldsymbol{\tau}_{A}\right]}{\arg\max}} \left|C\left(\boldsymbol{\varphi}_{D},\boldsymbol{\varphi}_{A},\boldsymbol{\tau}_{A},\left\{\boldsymbol{h}^{s}\right\}^{S}_{s=1}\right)\right|  $$

The contribution of the SMC can be expressed by the superposition of several individual specular propagation paths. As such, it becomes apparent to maximize the correlation function sequentially with respect to the parameters ***θ***_smc,*p*_. This implies that we can minimize the objective function in Eq. (21) sequentially with respect to the different parameter subsets. This sequential optimization for all the geometrical propagation parameters can be written as follows:

30 $$ \hat{\tau_{A}}_{,p} = {\underset{\tau_{A}}{\arg\max}} \left|C\left(\varphi_{D,p},\varphi_{A,p},\tau_{A},\left\{\boldsymbol{h}^{s}\right\}^{S}_{s=1}\right)\right|,  $$

31 $$ \hat{\varphi_{D}}_{,p} = {\underset{\varphi_{D}}{\arg\max}} \left|C\left(\varphi_{D,p},\varphi_{A,p},\hat{\tau_{A}}_{,p},\left\{\boldsymbol{h}^{s}\right\}^{S}_{s=1}\right)\right|,  $$

32 $$ \hat{\varphi_{A}}_{,p} = {\underset{\varphi_{A}}{\arg\max}}\left|C\left(\hat{\varphi_{D}}_{,p},\varphi_{A,p},\hat{\tau_{A}}_{,p},\left\{\boldsymbol{h}^{s}\right\}^{S}_{s=1}\right)\right|.  $$

The corresponding specular power of this path can then be determined by inserting these ML parameter estimates $\left [\hat {\varphi _{D}}_{,p},\hat {\varphi _{A}}_{,p},\hat {\tau _{A}}_{,p}\right ]$ into Eq. (24). The total specular path can then be described as follows:

33 $$ {} \hat{\boldsymbol{x}}^{s}_{p}\left(\hat{\boldsymbol{\theta}}^{s}_{\text{smc},p}\right) = \texttt{vec} \left(\boldsymbol{B}_{T}^{s}\left(\hat{\varphi_{D}}_{,p}\right) \Diamond \boldsymbol{B}_{R}^{s}\left(\hat{\varphi_{A}}_{,p}\right) \Diamond \boldsymbol{B}_{f}\left(\hat{\tau_{A}}_{,p}\right)\right) \hat{\gamma}^{s}_{p}.  $$

The initialization procedure as described above, i.e., finding the global ML estimates for the AoD, AoA, and ToA over UWB frequencies of a propagation path *p*, will subsequently be used for the estimation and optimization of the DMC and SMC parameter sets (see Fig. 1).

### Optimization of DMC and SMC

#### Estimation and optimization of DMC

After the initialization procedure (see Fig. 1), the UWB-RiMAX algorithm will estimate and optimize the DMC and noise parameter estimates in each sub-band $s \in \mathcal {S}$ separately by using a Gauss-Newton algorithm. This algorithm relies on the calculation of the correlation matrix of the residual signal (***h***−***x***(***θ***_smc_)), which is used to maximize the log-likelihood function with respect to the DMC parameters (see Eq. (20)). It uses the Jacobian of this log-likelihood function to update the DMC parameter set in a next iteration, until convergence of the result is achieved. Since no adjustments were made to the estimation and optimization algorithm of the DMC and noise parameters, we refer to [7] for its full mathematical description.

#### Estimation and optimization of SMC

**Fig. 2:**
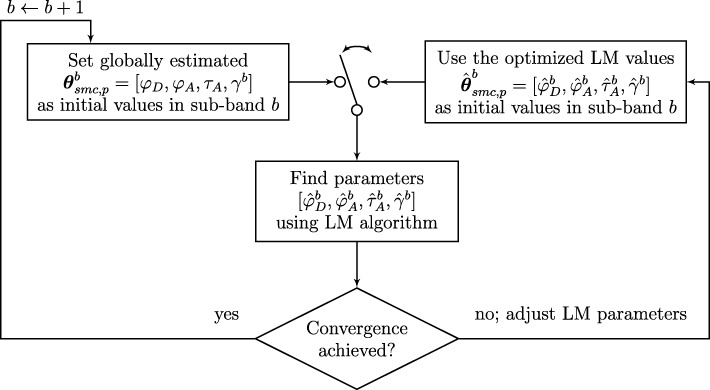
Flowchart of the estimation and optimization of the SMC. The first iteration of a new sub-band *s* will use the globally estimated SMC from the initialization procedure as described in Section 4.2.2 as initial values for the search of its optimal values. The switch ensures that subsequent iterations will use the previously optimized (but not converged) values for a better optimization. When convergence is achieved, the next sub-band *s*+1 will again use the globally estimated SMC as initial values for the optimization

The optimization of the SMC was performed as presented in Fig. 2. As we can see from this figure, the globally estimated geometrical parameters [*φ*_*D*_,*φ*_*A*_,*τ*_*A*_] over UWB frequency of each specular propagation path *p*, together with its frequency-dependent amplitude *γ*^*s*^ per sub-band $s \in \mathcal {S}$, are used as initial values for the search of their optimal values $\left [\hat {\varphi }^{s}_{D}, \hat {\varphi }^{s}_{A}, \hat {\tau }^{s}_{A}, \hat {\gamma }^{s}\right ]$ in sub-band *s*. By using the globally estimated geometrical parameters as initial values for the Levenberg-Marquardt (LM) algorithm [7], we can ensure that the optimal values in each sub-band lie close to these globally estimated values. By also making these geometrical parameters frequency-dependent per sub-band, we allow for more freedom in the estimation of the optimal value *γ*^*s*^ for the frequency-dependent amplitude. In the algorithm for the optimization of the SMC, the switch ensures that subsequent iterations will use the previously optimized (but not yet converged) SMC values for a better optimization. When eventually convergence is achieved, the next sub-band that is to be optimized will again use the globally estimated SMC as initial values for their optimization in sub-band *s*+1.

### Model order selection and the reliability of specular paths

#### Proposed method for model order selection

The total number of specular propagation paths *P* that is to be extracted from the measurement data is an issue that should be treated with care. This number will naturally influence the ratio between the total power of the SMC components and those that are categorized as DMC. Algorithms like the Akaike information criterion [51] or the minimum description length [52] can be used to tackle this problem. However, in this work, we will use the approach outlined in [7, 29] that is based on the estimated power of the extracted propagation paths. Because the RiMAX algorithm provides an estimate of the Fisher Information Matrix (FIM) as a by-product, the diagonal elements of the inverse of the FIM are estimates of the variance of the channel parameters in Eq. (5). For each estimated propagation path, it is possible to associate an SNR with it: a path *p* with an estimate $\hat {\gamma }_{p}$ for its complex amplitude, has an accompanying SNR $\hat {\rho }_{p}$ (dimensionless), equal to the following:

34 $$ \hat{\rho}_{p} = \frac{\left|\hat{\gamma}_{p}\right|^{2}}{\text{var}\left\{|\hat{\gamma}_{p}|\right\}}.  $$

In Eq. (34), var(·) denotes the variance of $\hat {\gamma }_{p}$. It can be proved that the $\left |\hat {\gamma }_{p}\right |$ estimator follows a half-normal distribution with variance $\text {var} \left (|\hat {\gamma }_{p}|\right)$ [7]. The SNR $\hat {\rho }_{p}$ in Eq. (34) then follows a chi-squared distribution with 2° of freedom $\left (\chi ^{2}_{2}\right)$. The propagation path *p* is considered to be unreliable and removed from further analysis if its estimated SNR is smaller than the 90 th percentile of $\chi ^{2}_{2}$, equal to 6.63 dB [29].

#### Stop criterion for path detection

In the flowchart of the UWB-RiMAX algorithm (see also Fig. 1), we can see that an attempt is made to extract more propagation paths from the measured (or residual) channel. It should be noted that (UWB-) RiMAX is an iterative algorithm, implying that in each iteration, it tries to estimate a fixed number of new specular paths from the measured (or residual) channel response. The number of new propagation paths per iteration was arbitrarily chosen as five, as originally proposed in [7]. It should be noted that this number can be chosen differently depending on the propagation environment. For example, indoor scenarios usually result in stronger multipath behavior than outdoor scenarios. If at least one of the five paths in an iteration succeeds the SNR threshold of 6.63 dB in all sub-bands, the algorithm keeps searching for new paths. Otherwise, the channel is considered to be exhausted of reliable specular paths, and the algorithm is stopped. Subsequently, the resulting SMC and DMC parameter sets are stored, after which they can be used for further analysis.

The model order selection based on Eq. (34) is more suited for this paper’s topic than a selection based on information criteria such as the Akaike one. The latter approach calculates an optimal value for the size of the signal subspace as a whole without deciding on the reliability of individual specular paths. In contrast, the path SNR method checks each individual path for its reliability. This approach is more in agreement with the philosophy of DMC, stating that they can also comprise specular paths which cannot be resolved reliably due to the limited apertures of the measurement equipment or the limited capabilities of the multipath estimation algorithm.

## Evaluation

### Synthetic radio channel modeling

In order to evaluate the UWB-RiMAX multipath estimation algorithm as described above, we have generated 200 synthetic channels with controlled parameters for the SMC and DMC parameters. In the following subsections, we first describe how these parameters were modeled in our evaluation procedure. Subsequently, we will describe how the estimated SMC and DMC are compared with their synthetically generated counterparts, in order to evaluate the performance of our algorithm.

#### Contribution of SMC

The generation of the SMC was performed by a ray-tracer in a virtual indoor environment of 15 m × 10 m × 3 m, where for each of the 200 channel realizations in total, random positions are chosen for the transmitter and receiver, with the restriction that they are at least 3 m separated from each other. The height of both transmitter and receiver was fixed at 1.5 m. Further details about the working of the ray-tracer are explained in [53]. For each channel realization, the ray-tracer launched several rays from the randomly positioned transmitter into the environment, each of which could undergo up to 7 reflections, until a total number of 40 paths were obtained at the receiver.

Both the transmitter and receiver consisted of a planar UCA with *M*_*T*_=*M*_*R*_=8 antennas, with an inter-element spacing of 0.45 times the wavelength at the highest UWB frequency (10.6 GHz), resulting in a diameter *d* (m) equal to the following:

35 $$ \begin{aligned} d &= \frac{0.45\times\lambda_{10.6~\text{GHz}}}{\sin(\pi/M_{T/R})} \\ &= 1.8~\text{cm} \end{aligned}  $$

The radio channel was then sampled at *M*_*f*_=4501 uniformly spaced frequency points ranging from 3.1 up to 10.6 GHz, resulting in a maximum measurable time-delay *τ*_max_ of 600 ns. An arbitrary sub-bandwidth of 250 MHz was assumed in the evaluation setup of the UWB-RiMAX algorithm, resulting in a total of 30 UWB frequency sub-bands in which the time-delay bin width *Δ*_*τ*_ is 4 ns.

#### Contribution of DMC and noise

In order to generate a realization of the DMC and noise process ***d***(***θ***_dan_) contributing to the radio channel, we first have to generate a circular Gaussian process with zero mean and a covariance matrix ***R***(***θ***_dan_) as follows:

36 $$ \boldsymbol{d}(\boldsymbol{\theta}_{\text{dan}}) \sim \mathcal{N}_{c} (\boldsymbol{0}, \boldsymbol{R}(\boldsymbol{\theta}_{\text{dan}})) \in \mathbb{C}^{M_{f} \times 1}.  $$

In order to do so, we will generate a multivariate i.i.d. circular Gaussian process ***z*** as follows:

37 $$ \boldsymbol{z} \sim \mathcal{N}_{c} (\boldsymbol{0}, \boldsymbol{I}) \in \mathbb{C}^{M_{f} \times 1},  $$

and use a transformation matrix ***L***(***θ***_dan_) satisfying:

38 $$ R(\boldsymbol{\theta}_{\text{dan}}) = \boldsymbol{L}(\boldsymbol{\theta}_{\text{dan}}) \cdot \boldsymbol{L}^{H}(\boldsymbol{\theta}_{\text{dan}}),  $$

so that we can finally compute ***d***(***θ***_dan_) as follows:

39 $$ \boldsymbol{d}(\boldsymbol{\theta}_{\text{dan}}) = \boldsymbol{L}(\boldsymbol{\theta}_{\text{dan}}) \cdot \boldsymbol{z}.  $$

In the equations above, the transformation matrix ***L***(***θ***_dan_) can be calculated by using the Cholesky decomposition or the singular value decomposition (SVD) of the covariance matrix ***R***(***θ***_dan_). Based on Eq. (11), we can construct this matrix by controlling the four parameters in ***θ***_dan_. The choice for *α*_0_ was chosen as the average noise-level of the SMC contribution to the channel, taking into account an SNR of 20 dB after calculating the average signal power of the SMC. Mathematically, *α*_0_ can be calculated as follows:

40 $$ \alpha_{0}|_{\text{dBm}} = P_{SMC}|_{\text{dBm}} - SNR|_{\text{dB}}.  $$

The value for *α*_1_ was chosen as the peak value of the SMC contributions, minus 3 dB. The reverberation time *τ*_rev_ was calculated based on Eq. (9), in which the coherence bandwidth *B*_*d*_ can be calculated from the root-mean-square (RMS) delay spread *τ*_RMS_ [s] as follows:

41 $$ B_{d} = \frac{1}{\tau_{\text{RMS}}},  $$

and *τ*_RMS_ can be calculated from the SMC power as follows:

42 $$ \tau_{\text{RMS}} = \sqrt{\frac{\sum\limits_{n} P_{\text{SMC}}(n)~\tau_{n}^{2}}{\sum\limits_{n} P_{\text{SMC}}(n)} - \left(\frac{\sum\limits_{n} P_{\text{SMC}}(n)~\tau_{n}}{\sum\limits_{n} P_{\text{SMC}}(n)}\right)^{2}},  $$

in which *τ*_*n*_ (s) is the time-delay of the *n*th delay-bin, which is equal to *n*×*Δ*_*τ*_, and *P*_SMC_(*n*) is the PDP of the SMC, which can be calculated by performing an Inverse Discrete Fourier Transform (IDFT) operation on the signal ***x***(***θ***_smc_). This DMC model was then superimposed onto the SMC contribution to the channel, which was generated with the help of the ray-tracer as described in Section 5.1.

### Pairing of estimated multipath parameters

In order to evaluate how well the synthetically generated SMC are estimated by the algorithm, we will make use of the multipath component distance (MCD) [54, 55]. The MCD (dimensionless) can be seen as a metric to define the “closeness” between two parameter sets in multipath parameter distance space. It was previously shown that it outperforms the Euclidean distance, and is a suitable metric for combining parameters that have different units (as is the case here with both angles and delays). The pairing of each UWB-RiMAX estimated SMC with its synthetically generated counterpart is done by searching for the smallest MCD between each (output) estimated SMC and the (input) synthetically generated SMC. The MCD $\Omega _{\varphi _{i,j}}$ (dimensionless) between two distinct angles *φ*_*i*_ and *φ*_*j*_, i.e., the angular distance between both, can be calculated as follows:

43 $$ \Omega_{\varphi_{i,j}} = \frac{1}{2} \left| \left(\begin{array}{l} \cos (\varphi_{i}) \\ \sin (\varphi_{i}) \\ \end{array}\right) - \left(\begin{array}{l} \cos (\varphi_{j}) \\ \sin (\varphi_{j}) \\ \end{array}\right)\right|  $$

The MCD $\Omega _{\tau _{i,j}}$ (dimensionless) between two distinct angles time-delay instances *τ*_*i*_ and *τ*_*j*_ can be written as follows:

44 $$ \Omega_{\tau_{i,j}} = \zeta \cdot \frac{|\tau_{i}-\tau_{j}|}{\Delta \tau_{\text{max}}} \cdot \frac{\tau_{std}}{\Delta \tau_{\text{max}}}  $$

with *τ*_std_ being the standard deviation of all delays *τ*, and *Δτ*_max_ calculated as follows:

45 $$ \Delta \tau_{\text{max}} = \max_{i,j}\left\{\left|\tau_{i}-\tau_{j}\right|\right\}.  $$

The parameter *ζ* is a delay scaling factor to give the delay more weight in the MCD metric when necessary. This factor was chosen as 1, as originally proposed in [55]. Higher values can be chosen to give more weighting to the delay for the pairing of multipath parameters. The delay distance of the MCD was scaled with the normalized delay spread $\frac {\tau _{\text {std}}}{\Delta \tau _{\text {max}}}$. The resulting MCD metric *Ω*_*i*,*j*_ between two multipath parameter sets *i* and *j* can then be calculated as follows:

46 $$ \Omega_{i,j} = \sqrt{\Omega_{\varphi_{D_{i,j}}}^{2} + \Omega_{\varphi_{A_{i,j}}}^{2} + \Omega_{\tau_{i,j}}^{2}},  $$

which can be interpreted as the radius of a circle in the normalized multipath parameter distance space.

It should be noted that in our scenario, there is no one-to-one matching of the (output) estimated SMC to the (input) synthetically generated SMC. The fact that we allow multiple output SMCs to be matched to the same input SMC is to overcome the case when, e.g., the remainder of the signal after subtraction of previously detected paths at a certain time-delay instance, still contains a sufficient amount of power at this time-delay instance. Newly detected paths then have the same time-delay value and directional parameters, so that we allow them to be matched to the same input SMC.

## Results of simulations

**Fig. 3:**
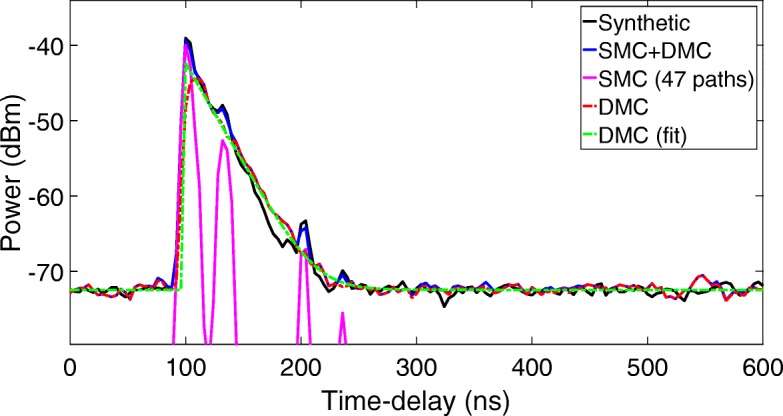
APDP of the synthetic radio channel in the time-delay domain, together with the UWB-RiMAX retained SMC, the DMC, and noise

Figure 3 shows the averaged power delay profile (APDP) in the time-delay domain for a randomly generated synthetic radio channel as described in the previous chapter, as well as the UWB-RiMAX estimated SMC, the residual DMC, and noise, and its exponential fit from Eq. (6). Looking at this figure, we can state that the SMC components are well estimated from the radio channel and that the DMC and noise exponential decay (linear in a dBm-scale) of the radio channel is clearly visible and perfectly estimated from the simulations.

### Estimation of propagation paths

#### MCD metric

**Fig. 4:**
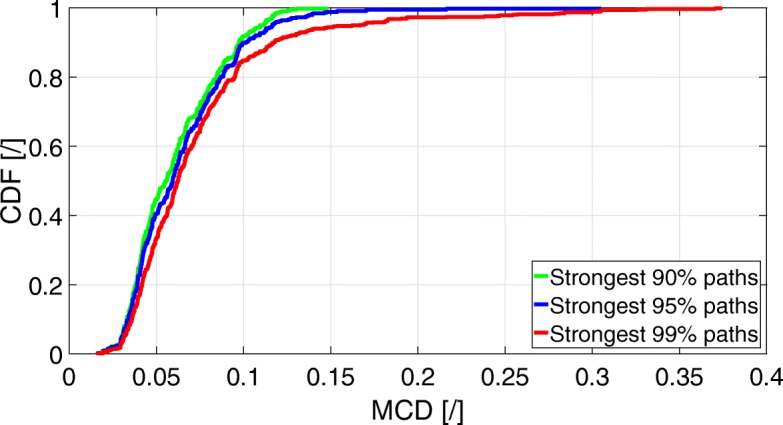
CDF of the MCD metrics between the input and output strongest 90, 95, and 99% propagation paths

Figure 4 shows the cumulative distribution function (CDF) of the resulting MCDs between the 90, 95, and 99% strongest input paths, and their closest output path in multipath parameter space to the inputs. Table 1 shows the 5th, 50th (median), and 95th percentiles of the MCD metric as a function of the percentage of strongest input paths.

**Table 1:** Percentiles of the MCD metric as a function of the number of strongest input propagation paths

Number of strongest input paths (%)	MCD metric [/] - percentiles		
	5th	50th	95th
90	0.0227	0.0443	0.1066
95	0.0228	0.0477	0.1136
99	0.0235	0.0554	0.1404

Figure 4 and Table 1 show that when taking into account the 90 or 95% strongest input paths, at least 95% of those are closely matched with the output paths when applying a 0.11 threshold for the MCD metric. When taking into account the 99% strongest input paths, at least 95% of those are closely matched with the output paths when applying a 0.14 threshold for the MCD metric. Since the MCD is a bounded dimensionless metric between 0 and 1, we can safely state that 99% of the strongest input paths are very well estimated by our algorithm.

#### Angular differences

**Fig. 5:**
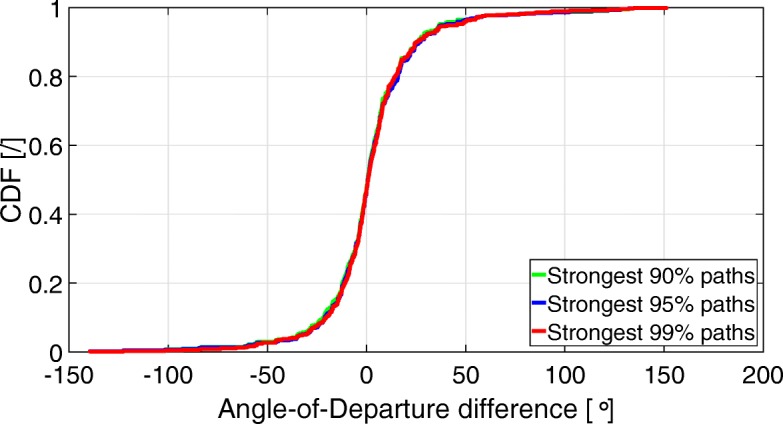
CDF of AoD differences between the input and output strongest 90, 95, and 99% propagation paths

**Fig. 6:**
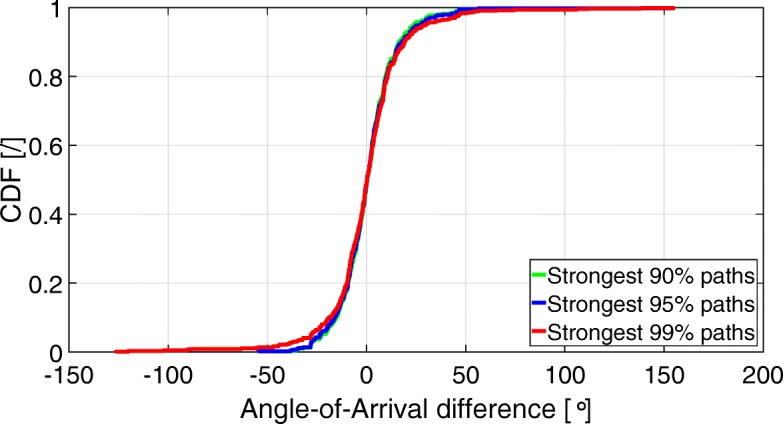
CDF of AoA differences between the input and output strongest 90, 95, and 99% propagation paths

Figures 5 and 6 show the CDF of the angular differences (AoD and AoA) between the 90, 95, and 99% strongest input paths, and their closest output path. Table 2 shows the 5th, 50th (median), and 95th percentiles of the angular differences as a function of the percentage of strongest input paths.

**Table 2:** Percentiles of the angular differences (AoD and AoA) as a function of the number of strongest input propagation paths

Number of strongest input paths		Angular difference [°] - percentiles		
		5th	50th	95th
90%	AoD	– 30.47	0.96	35.64
	AoA	– 22.17	0.27	24.48
95%	AoD	– 29.08	0.98	37.52
	AoA	– 22.75	0.27	25.13
99%	AoD	– 29.24	1.01	44.12
	AoA	– 27.65	0.27	26.76

Figures 5 and 6 and Table 2 show that when taking into account the 99% strongest input paths, 90% of them have an AoD error somewhere between − 29.24° and + 44.12°, and an AoA error somewhere between − 27.65° and + 26.76°. Table 2 also shows that the median error (50th percentile) is smaller or equal to 1°, regardless of whether it is the AoD or AoA. The table also shows that the range between the 5 and 95th percentiles, so where 90% of the estimated values lie between, gets larger when taking into account more input paths. For example, when considering the 90% strongest input paths, the difference between the 5 and 95th percentiles AoA error is 46.65°, where it is 47.88° when considering the 95% strongest input paths, and 54.41° when considering the 99% strongest input paths.

#### ToA differences

**Fig. 7:**
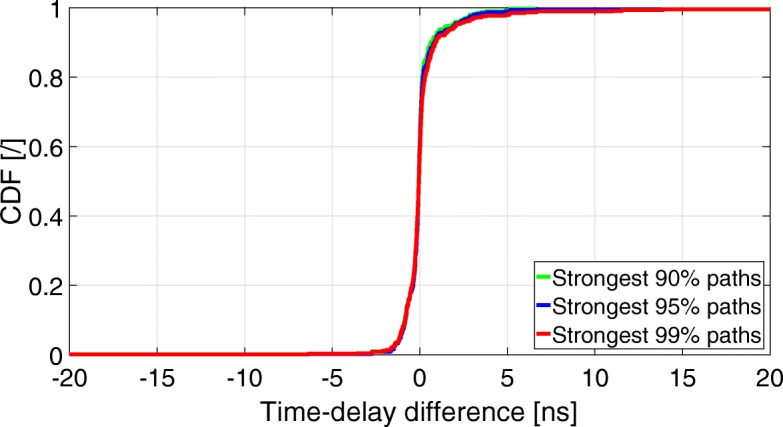
CDF of the ToA differences between the input and output strongest 90, 95, and 99% propagation paths

Figure 7 shows the CDF of the ToA differences between the 90, 95, and 99% strongest input paths, and their closest output path. Table 3 shows the 5th, 50th (median), and 95th percentiles of the ToA differences as a function of the percentage of strongest input paths.

**Table 3:** Percentiles of the ToA differences as a function of the number of strongest input propagation paths

Number of strongest input paths (%)	ToA difference (ns) - percentiles		
	5th	50th	95th
90	– 0.81	0.02	7.51
95	– 0.81	0.02	7.49
99	– 1.12	0.01	6.69

Figure 7 and Table 3 show that when taking into account the 99% strongest input paths, 90% of them have a ToA error somewhere between − 1.12 and + 6.69 ns. Table 3 also shows that the median error (50th percentile) is around 0.02 ns, regardless of whether we take the 90, 95, or 99% strongest input paths.

### Estimation of reverberation times

**Fig. 8:**
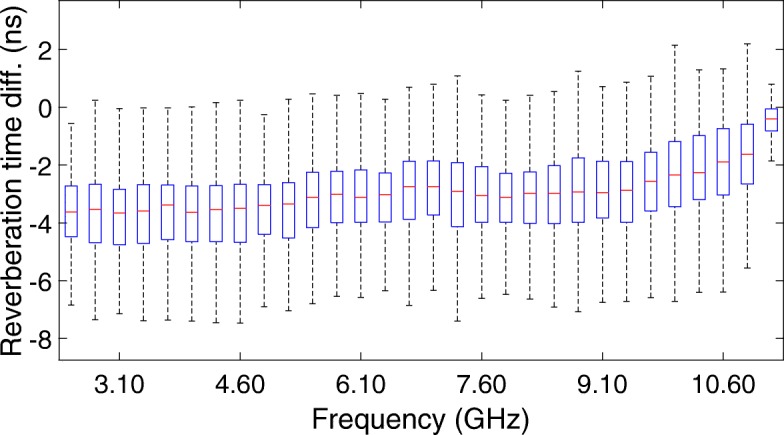
Box plot of the differences between the input and output reverberation time

All synthetically generated propagation paths in the APDP were superimposed with a DMC model, as described in Section 5.1.2. Figure 8 shows the resulting differences between all generated and estimated reverberation times as a function of UWB sub-band. This data is represented by means of box plots per frequency band, which indicate the first, second (median), and third quartiles of these differences, and the 1.5 interquartile range from both the lower and upper quartiles.

Figure 8 shows that a median error of less than 4 ns can be obtained for the lower frequencies in the UWB band. For the higher frequencies in the UWB band, a median error of less than 2 ns can be obtained. Overall, more than 75% of our simulation results show an absolute difference between true and estimated reverberation times of less than 4.6 ns in the worst-case scenario, which are more than acceptable results given the relatively low SNR of 20 dB in our evaluation.

### Estimation of signal powers

**Fig. 9:**
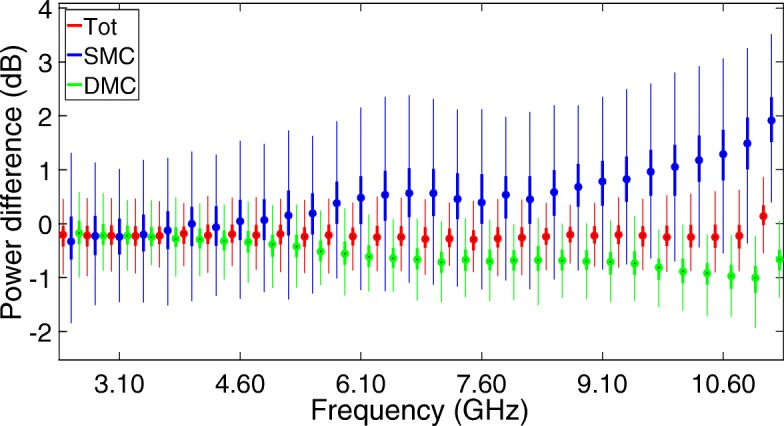
Box plot of the differences between the input and output powers for the total channel, as well as the contributions of both the SMC and DMC power

Figure 9 depicts the box plots of the difference between the input and output powers for the total channel, as well as the contributions of both the SMC and DMC power.

From Fig. 9, we can see that the total power in the channel is very well estimated by the UWB-RiMAX algorithm, both at the lower and higher frequencies. We can also observe median differences between the true and estimated SMC powers ranging between –0.3 dB at the lower UWB frequencies, increasing to about 1.5 dB at the higher UWB frequencies. These are more than acceptable errors in the estimation of the SMC signal power, if we take into account that the error is still less than 1 dB up to 9 GHz. Next to that, we can observe median absolute differences between the true and estimated DMC powers of about –0.2 dB at the lower UWB frequencies, increasing to about –1 dB at the higher UWB frequencies. The fact that the power of both SMC and DMC is estimated slightly worse at the higher UWB frequencies might be resolved by softening the stop criterion at these frequencies, as it is currently influenced by the SNR of each path, which is thus lower at higher frequencies than at lower frequencies.

Overall, the values for the MCD metric show that our proposed algorithm is able to correctly estimate the most significant input propagation paths, and by analyzing the differences between the input and output SMC, DMC, and total powers, we can state that our proposed algorithm gives a fairly good agreement between the generated input values and the estimated output values.

## Results of measurements

### Measurement scenario

#### Measurement environment

**Fig. 10:**
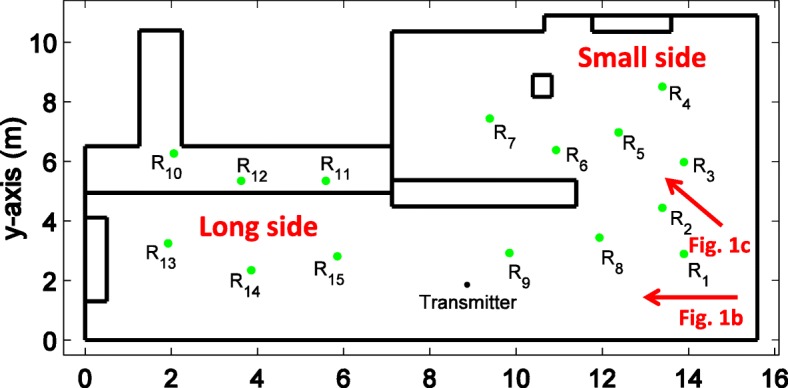
Measurement environment: schematic representation

**Fig. 11:**
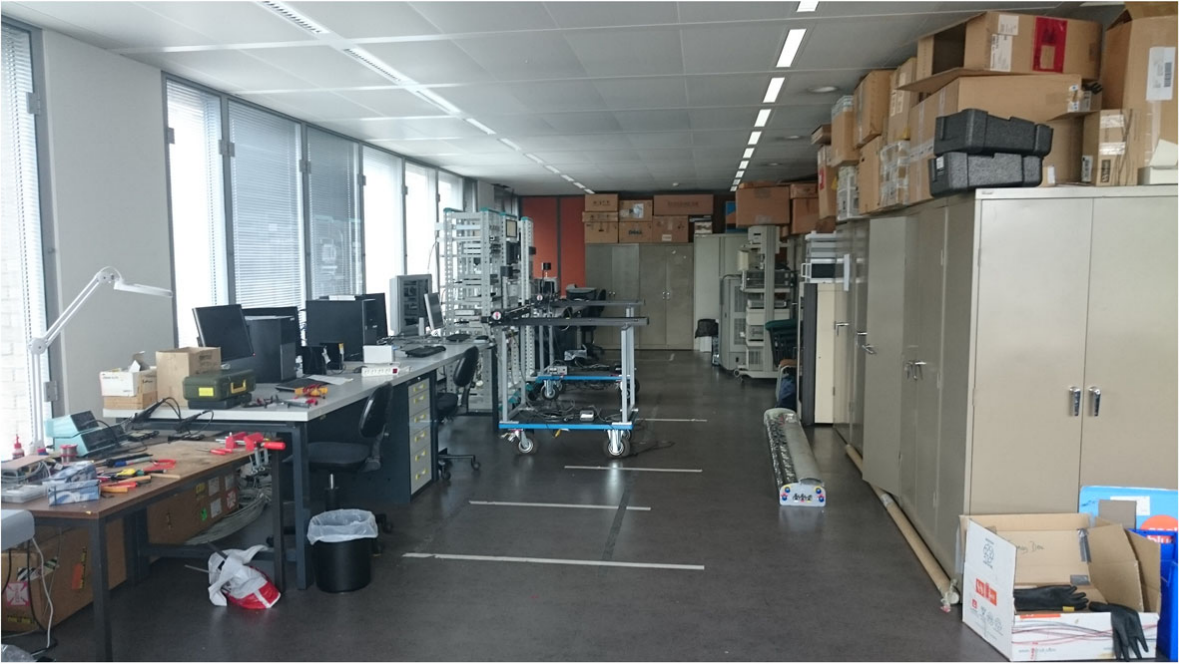
Measurement environment: photograph of long side

**Fig. 12:**
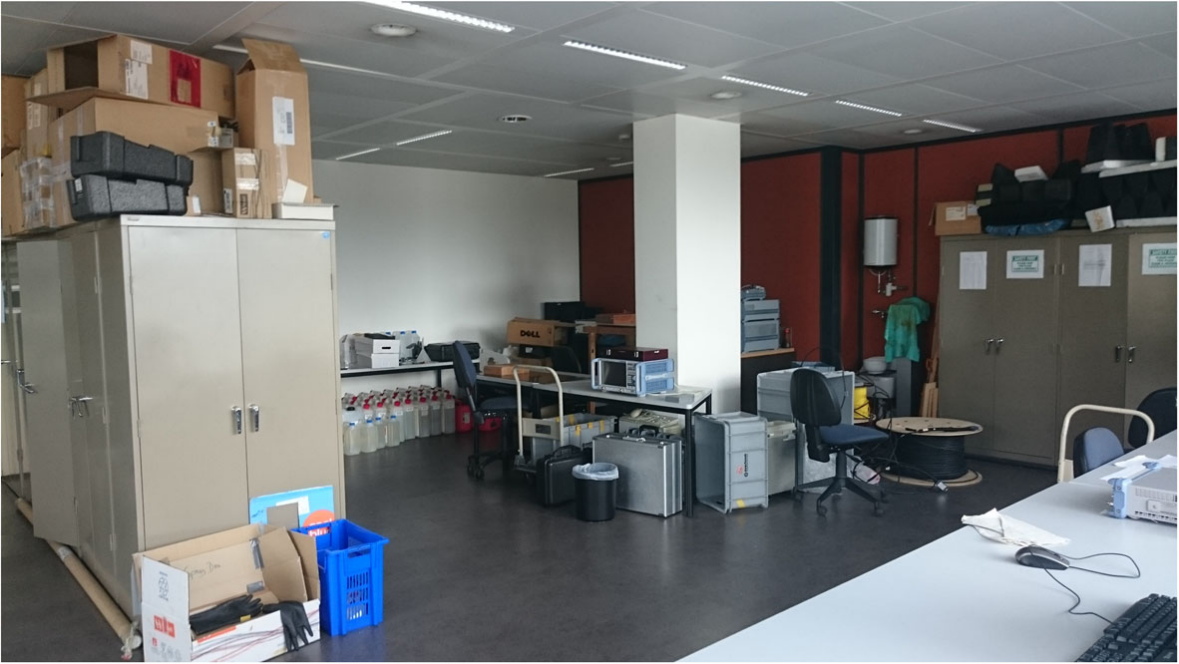
Measurement environment: photograph of small side

In order to test our UWB-RiMAX algorithm, we have performed indoor measurements in a laboratory of Ghent University in Belgium. The schematic representation of the measurement environment is depicted in Fig. 10, with an indication of what we call the long and small side of the laboratory. The long side was approximately 16 m long and 5 m wide (see Fig. 11), and the small side adjacent to it was approximately 8.5 m long and 5 m wide (see Fig. 12). In this environment, most of the equipment consisted of metallic cabinets, tables, computers, and various other hardware. As can be seen from the pictures below, the environment was a very cluttered one.

In this laboratory environment, the indoor radio channel was measured between 15 spatially distinct receiver positions with respect to one single transmitter (see Fig. 10 for an outline of their positions). The exact positions of the receivers with respect to the transmitter were measured by means of a digital laser distance meter, which had an accuracy of 2 mm. In total, 8 of the 15 positions were considered LoS scenarios, where there is a direct (free space) path from the transmitter to the receiver. Next to that, 4 of the 15 positions were considered OLoS scenarios, where the free space path from transmitter to the receiver can only be reached via a reflection and/or a diffraction. Lastly, 3 of the 15 positions were considered non-line-of-sight (NLoS) scenarios, where the path from the transmitter to the receiver needs to undergo a transmission through a certain medium (in this case, a plasterboard wall).

#### Channel sounding procedure

**Fig. 13:**
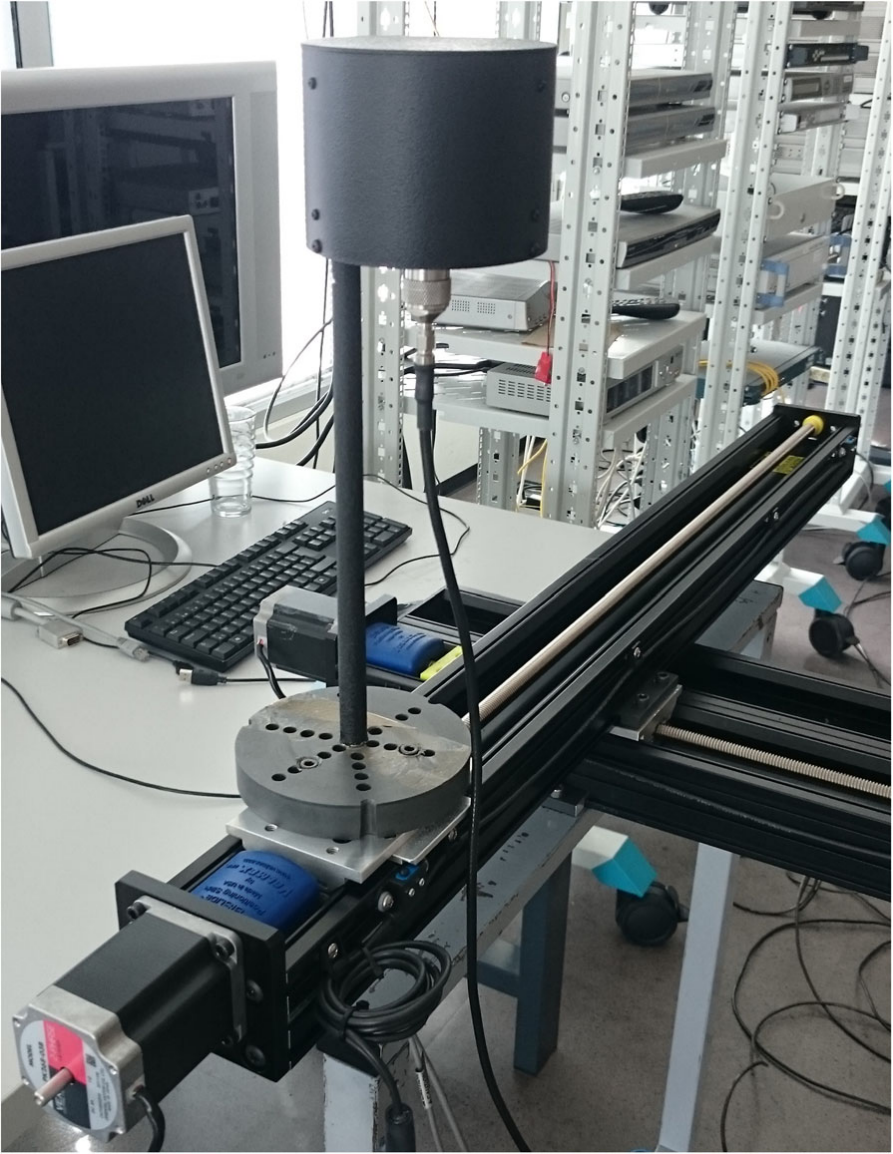
Automated positioning system and UWB antenna, to form a planar horizontal virtual UCA

At each of the 15 indoor positions, we have carried out channel sounding measurements with a vector network analyzer (VNA) of type Rohde & Schwarz ZNB8 to probe the radio channel ranging from 3.1 up to 10.6 GHz. A planar horizontal virtual [8×8] UCA was created at the transmit and receive side of the measurement system by an automatic positioning system (see Fig. 13). The UCA is preferred over other planar array configurations due to its circular configuration, through which it has a 360° full azimuthal field of view (in contrast to linear arrays). A virtual array ensures that the antennas do not suffer from mutual coupling. The inter-element spacing between two adjacent antennas on the virtual UCA was 0.45×*λ*_10.6 GHz_=1.27 cm. Both antennas were omni-directional UWB antennas in the azimuthal plane of type Electro-Metrics EM-6865 [56], placed 1.5 m above the ground.

In the 3.1 to 10.6 GHz UWB frequency band, the VNA sampled the complex gain between each pair of Tx and Rx antennas over *M*_*f*_=7501 uniformly spaced frequency point, with a resolution bandwidth of 10 kHz. This complex gain corresponds with the *S*_21_-scattering parameter, which is the ratio of the output reflected power wave divided by the input incident power wave, where all values are expressed as complex quantities. Our measurements resulted in a maximum measurable time-delay *τ*_max_ of 1000 ns. The cables connecting Tx and Rx antennas were included in the calibration of the VNA to exclude their influence from the measurement data. Measurements were conducted outside of working hours to get a static radio channel without any movement.

#### Frequency stationarity

Prior to the processing of the measurement data in the UWB band, we will first check the uncorrelated scattering (US) assumption which is often assumed for the modeling of wireless channels. The US assumption states that the second-order statistical description of the radio channel is independent of the absolute frequency, which implies that channel contributions at different time-delays are uncorrelated. In order to evaluate the US assumption in the UWB band, we will apply the procedure explained in [57], which proposes a test that defines a frequency stationarity region (or stationarity bandwidth) in which the US assumption holds. This test is based on the definition of a minimum stationarity region, which is a power spectral density in the frequency domain in which the US property locally holds. Subsequently, the test measures how many neighboring MSRs can be formed, of which their adjacent overlap in the frequency domain exceeds a certain threshold. The total number of neighboring MSRs which exceed this threshold thus form a frequency stationarity region, corresponding with a certain number of frequency samples (and thus a certain bandwidth).

**Fig. 14:**
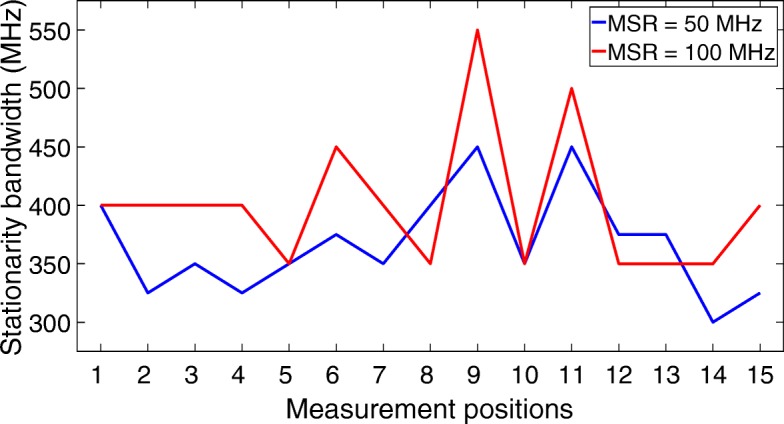
Analysis of the stationarity bandwidth of the measurement data for different values of the MSR

We have analyzed the stationarity bandwidths for the different measurement positions for MSR values of 50 MHz (corresponding with 50 frequency samples) and 100 MHz (corresponding with 100 frequency samples) with a threshold value of 0.9 to decide if neighboring MSRs can be considered stationary in the frequency domain. Choosing a smaller value for the MSR would deteriorate its resolution. The results of this analysis can be found in Fig. 14.

Figure 14 shows that it is reasonable to assume a sub-bandwidth of 250 MHz in the setup of the UWB-RiMAX algorithm, in which we can thus state that the US assumption holds. This analysis proves that it is even reasonable to assume sub-bandwidths of 300 MHz.

### Results of SMC and DMC

**Fig. 15:**
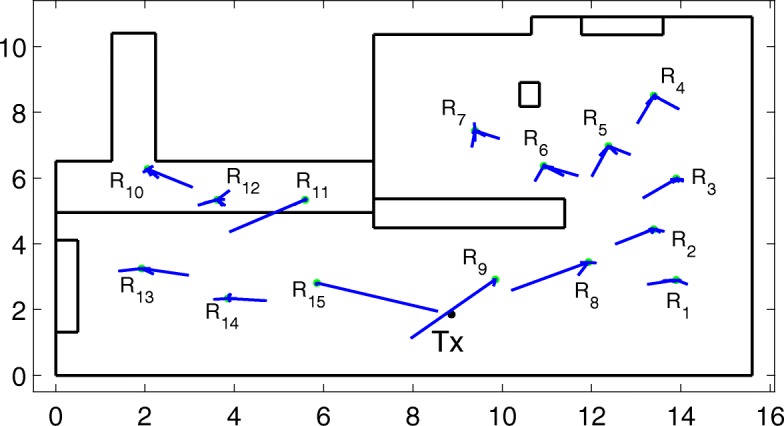
Estimated geometrical propagation paths in the environment

Figure 15 shows the 10 strongest estimated geometrical propagation paths in the environment. Their length is an indication of their relative power.

From Fig. 15, we can see that our algorithm is able to estimate the correct geometrical propagation paths in the environment. For receiver positions 11 and 12, it looks like the algorithm estimated the wrong angles, but it should be noted that there was a metallic cabinet in the long side of the environment on which the paths apparently scattered from transmitter to receiver.

**Fig. 16:**
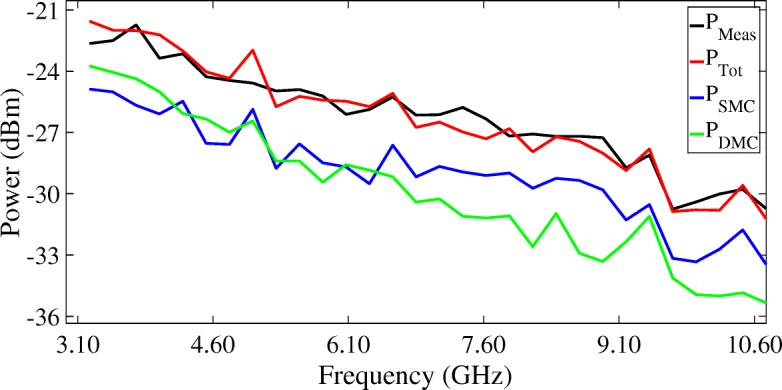
Mean values of the measured powers in the radio channel as a function of UWB frequencies: LoS scenario

**Fig. 17:**
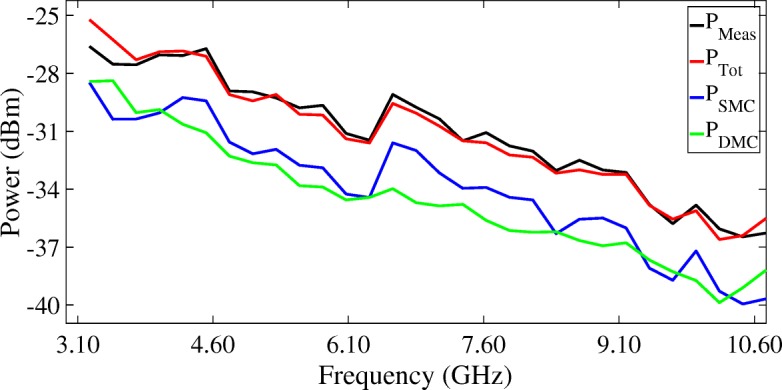
Mean values of the measured powers in the radio channel as a function of UWB frequencies: OLoS scenario

**Fig. 18:**
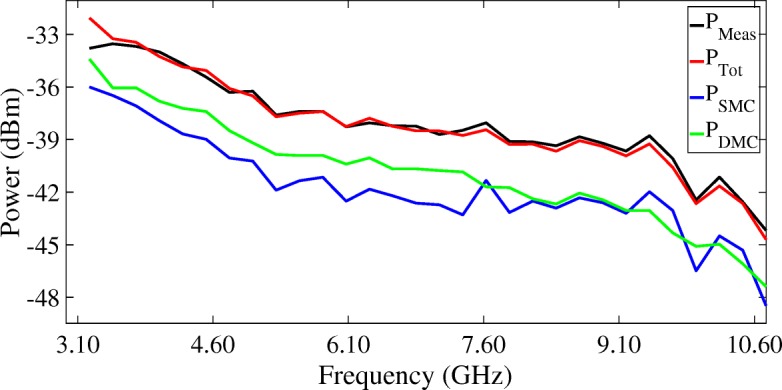
Mean values of the measured powers in the radio channel as a function of UWB frequencies: NLoS scenario

Figures 16, 17, and 18 show the mean values of the measured SMC, DMC, and SMC+DMC (total reconstructed) powers in the radio channel as a function of UWB frequencies. The power of the SMC part of the radio channel in each sub-band $P^{s}_{SMC}$ can be calculated from Eq. (19), whilst the power of the DMC part of the radio channel in each sub-band $P^{s}_{DMC}$ can be calculated from Eq. (39). The total reconstructed power in each sub-band $P^{s}_{Tot}$ can then be calculated by summing over both $P^{s}_{\text {SMC}}$ and $P^{s}_{\text {DMC}}$. Figure 16 presents the results of the LoS scenarios, Fig. 17 presents the results of the OLoS scenarios, and Fig. 18 presents the results of the NLoS scenarios.

Figures 16, 17, and 18 show that the SMC+DMC (total reconstructed) powers match very well with the measured power in the channel across the UWB frequency band. For the LoS scenario, the difference between the measured and the reconstructed power resulted in a minimum (underestimated) value of –1.61 dB and a maximum (overestimated) value of 1.20 dB. For the OLoS scenario, the minimum difference was –1.38 dB, and the maximum difference was 0.55 dB. For the NLoS scenario, the minimum difference was –1.73 dB, and the maximum difference was 0.52 dB. Figure 16 shows that the SMC power dominates the DMC power in a LoS scenario, especially for the higher UWB frequencies. This can be explained by the fact for higher frequencies, the wavelength of the transmitted ray is small, meaning that an incident ray on a surface will encounter little effect from the roughness of the material and will reflect on it specularly. In an OLoS scenario, Fig. 17 shows that both the SMC and DMC power result in comparable power levels. This can be explained by the fact that the inherent necessity of a reflection from transmitter to receiver will automatically generate more DMC in the channel, originating from the roughness of the surface on which the reflection of the path occurs. The more reflections a propagation path undergoes, the higher the chance that the incident wave at the receiver will have encountered diffuse scattering along the way. In an NLoS scenario, Fig. 18 shows that the DMC power dominates the SMC power, especially for the lower UWB frequencies. This can be explained by the fact for lower frequencies, the wavelength of the transmitted ray is large, meaning that an incident ray on a surface with irregularities comparable in size to its wavelength will cause this ray to be scattered at many angles rather than just at one angle (as is the case with a specular reflection). This diffuse scattering typically occurs at lower frequencies, which explains why the contribution of the DMC is significantly higher than those of the SMC. The DMC power ratio will be discussed in the next section.

### DMC power ratio

The DMC power ratio $p^{s}_{r}$ can be quantified as the relative power attributable to the DMC part of the measured radio channel and can be written as follows in each sub-band *s*:

47 $$ p^{s}_{r} = \frac{\sum\limits_{n=1}^{M_{f}}P^{s}_{\text{DMC}}(n)}{\sum\limits_{n=1}^{M_{f}}P^{s}_{\text{Meas}}(n)}.  $$

**Fig. 19:**
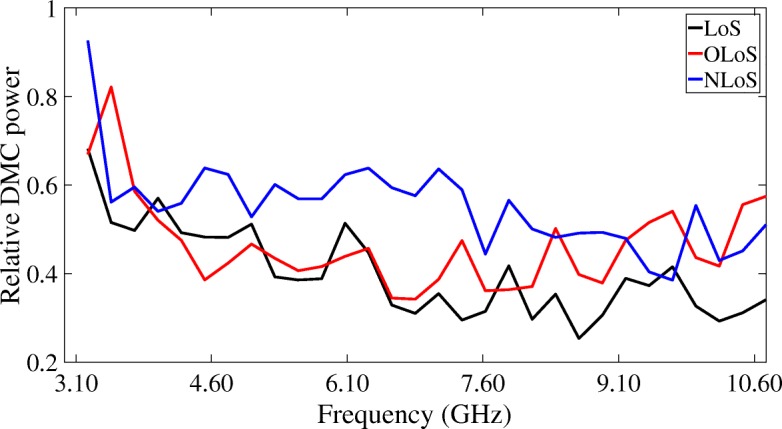
Relative DMC power ratio *p*_*r*_ as a function of UWB frequencies for LoS, OLoS, and NLoS scenarios

Figure 19 shows that the relative power attributable to the DMC part of the measured radio channel is higher for NLoS scenarios than for OLoS and LoS scenarios. In a LoS scenario, the presence of a strong direct path (LoS component) will dominate in power over the reflected paths, such that the relative DMC power ratio is lower than for OLoS or NLoS scenarios. The DMC power represents up to 50% of the total measured power for the lower UWB frequencies down to 30% for the higher UWB frequencies. As in the previous section, we know that this is due to the fact that an incident ray on an electrically small surface will encounter more effect from the roughness of its material, causing it to reflect on it diffusely. In contrast, in an OLoS scenario, the path between transmitter and receiver has to undergo one or more reflections, giving rise to more diffuse scattering along the way. This effect can be especially seen around 3 to 4 GHz. Finally, in an NLoS scenario, the DMC power represents up to 60% of the total measured power for the lower UWB frequencies (3.1 to 7 GHz), whilst it still represents up to 50% of the total measured power for the higher UWB frequencies (7 to 10.6 GHz). The necessity of a transmission from Tx to Rx in these NLoS scenarios between different media gives rise to more diffuse behavior of the waves, caused by the inherent roughness of the surfaces which the wave has to pass through. Overall, we can see that the DMC power ratio is lower for these higher frequencies, due to the fact that the encountered surfaces from Tx to Rx are electrically larger, resulting in more specular reflections.

## Conclusions

This work presented an extension of the RiMAX multipath estimation algorithm, facilitating the analysis of frequency-dependent propagation parameters for ultra-wideband (UWB) channel modeling. The newly proposed algorithm is capable of tracking both the specular and dense multipath components (SMC and DMC) over different UWB sub-bands by estimating the most likely geometrical propagation parameters such as angle of departure (AoD), angle of arrival (AoA), and time-delay of arrival (ToA) of the SMC occurring throughout these bands. In order to do so, a maximum likelihood (ML) criterion was proposed, allowing to estimate those propagation paths representing a significant amount of power in the measured radio channel over all sub-bands. The model for the DMC and noise was based on the observation that its power in the time-delay domain follows an exponential decay, and is related to the distance between transmitter and receiver. This algorithm was tested by generating synthetic radio channels in an indoor environment, with contributions to these channel consisting of SMC generated with a ray-tracer, and an associated DMC model. The geometric parameters were estimated from these synthetic models and matched with the generated parameter values with the help of the multipath component distance (MCD) metric. This metric was also used to define the closeness between the generated and estimated parameters in multipath parameter distance space. Next to that, the powers of the reconstructed SMC, DMC, and total channel were compared with their generated values.

Our simulation results show that the newly designed UWB-RiMAX algorithm can match up to 99% of the SMC parameters according to the MCD metric, and that the DMC reverberation time known from the theory of room electromagnetics can be estimated on average with an error margin of less than 2 ns throughout the UWB frequency band.

Our measurement results indicate the strong presence of DMC in an indoor environment, in which the DMC power represents up to 50% of the total measured power for the lower UWB frequencies, caused by the fact that the encountered surfaces from Tx to Rx are electrically smaller, resulting in more diffuse reflections. This DMC power ratio reduces to around 30% for the higher UWB frequencies since these surfaces will react more as specular reflectors.

Future work consists of performing a more extensive measurement campaign in line-of-sight (LoS), obstructed-LoS and non-LoS environments, and the estimation of SMC and DMC parameters in these different scenarios. The results from this measurement campaign will be used in a localization algorithm in order to estimate the location of a mobile receiver node with the help of a single transmitter node.

## Abbreviations

AoA: Angle of arrival
AoD: Angle of departure
APDP: Averaged power delay profile
BLUE: Best linear unbiased estimate
CDF: Cumulative distribution function
DMC: Dense multipath components
ESPRIT: Estimation of signal parameters via rotational invariance techniques
FIM: Fisher information matrix
GBSCM: Geometry-based stochastic channel model
IDFT: Inverse discrete fourier transform
i.i.d.: Independent and identically distributed
LM: Levenberg-Marquardt
LoS: Line-of-sight
MCD: Multipath component distance
MIMO: Multiple-input multiple-output
ML: Maximum likelihood
MSR: Minimum stationarity region
NLoS: Non-line-of-sight
OLoS: Obstructed-line-of-sight
PAP: Power angular profile
PDP: Power delay profile
RMS: Root-mean-square
Rx: Receiver
SAGE: Space-alternating generalized expectation-maximization
SMC: Specular multipath components
SNR: Signal-to-noise ratio
SVD: Singular value decomposition
ToA: Time-delay of arrival
Tx: Transmitter
UCA: Uniform circular array
US: Uncorrelated scattering
UWB: Ultra-wideband
VNA: Vector network analyzer
WPAN: Wireless personal area network
